# Waiting time and the psychosocial consequences of false-positive mammography: cohort study

**DOI:** 10.1186/s12952-015-0028-6

**Published:** 2015-04-30

**Authors:** Bruno Heleno, Volkert Siersma, John Brodersen

**Affiliations:** Department of Public Health, The Research Unit for General Practice and Section of General Practice, University of Copenhagen, Øster Farimagsgade 5, post-box 2099, 1014 Copenhagen K, Denmark

## Abstract

**Background:**

There is wide variation in the psychosocial response to false-positive mammography. We aimed to assess whether women having to wait longer to exclude cancer had increased psychosocial consequences that persisted after cancer was ruled out.

**Findings:**

We selected women with false-positive mammography (n = 272), screened for breast cancer in Copenhagen and Funen (Denmark) over a 1-year period. We measured psychosocial consequences immediately before women attended their recall visit and 1, 6, 18 and 36 months after women received their final diagnosis. After women were told that cancer had been ruled out, adverse psychosocial consequences decreased with time. We found no statistically significant differences between women who had cancer ruled out immediately at the recall visit (waiting time of 0) and women who had to wait longer before cancer was ruled out (waiting times 1-30, 30-120 and > 120 days), when psychosocial consequences were measured via a condition-specific questionnaire (Consequences of Screening in Breast Cancer) at 5 time points (0, 1, 6, 18 and 36 months after cancer exclusion).

**Conclusion:**

We did not confirm that waiting time was associated with worse long-term psychosocial consequences but type II error (failure to detect a true difference) might be a plausible explanation for our results.

## Introduction

We have shown in a cohort of women screened with mammography and followed-up for 36 months that false-positives were associated with negative long-term psychosocial consequences [1]. Moreover, in the same cohort, we have shown the invasiveness of the downstream procedures does not influence the degree of psychosocial consequences: women having non-invasive procedures suffers the same degree of negative consequences as those having invasive procedures [2]. Yet, there is wide variation in the psychosocial response to false-positives and it would be clinically relevant to find predictors of increased psychosocial harm [3-8].

In the critical period between positive screening test and the final diagnostic test that excludes cancer, women often think that they have a fatal disease [9,10]. This period may be particularly damaging in women caught up in an endless cycle of testing [11], i.e. women who keep having unclear results and who are asked to repeat diagnostic tests over a period of weeks or months. Thus, in our cohort, we now wanted to assess whether women having to wait longer for cancer exclusion had increased adverse psychosocial consequences that persisted after cancer was ruled out.

## Materials and methods

This paper is a post-hoc subgroup analysis of an earlier fixed cohort study [1]. We selected women with false-positive mammography, which enrolled women aged 50 to 69 years in two mammography screening programmes (Copenhagen and Funen, Denmark) between June 3, 2004 and June 2, 2005. Details of the original cohort have been previously reported [1].

The exposure of interest (waiting time until cancer exclusion) was calculated from the administrative records as the period between the day women called to book an appointment after receiving the recall letter and the date of final diagnosis. We did not expect waiting time to have a linear effect in psychosocial consequences. We thought that the critical difference would be between receiving the diagnosis in the same day and having to wait until getting the diagnosis [12]. Also, an earlier study about anxiety related to mammography screening found a non-linear relationship between anxiety and time until the first diagnostic test [13]. To address the possibility of non-linear effects waiting time was categorised. Because we could not find literature to guide the choice of categories, we used the following arbitrary cut-offs: 0 days, 1-30, 30-120 and > 120 days.

The main outcome was psychosocial consequences of screening. It was assessed through a condition-specific questionnaire – the Consequences of Screening in Breast Cancer (COS-BC) [14-16]. We used the sum-score of all 29 items in part-1 of the COS-BC. This score is supported by a confirmatory factor analysis [15] and was used in an earlier study [2]. The outcome was assessed at 5 points in time: women were invited to complete the questionnaire at the recall clinic (immediately before their consultation) and at 1, 6, 18 and 36 months after cancer had been excluded [1].

We have collected data on four potential confounders with the first questionnaire: age, social class, employment, and whether the woman lived alone. Age was treated as a continuous variable.

The mean score for each outcome throughout time was analysed using linear regression models. We used both crude models and models adjusted for the four potential confounders. We defined significance at P < 0.05. Scales set to missing were not included in the analyses. All analyses were performed with SAS 9.3 (SAS Institute, Inc., Cary, NC).

The study was approved by the Danish Data Protection Agency, 2007-41-0777. Approval from the ethics committee was not required. In accordance to Danish law, assessment by an ethics committee is only required when new interventions are being tested or if biological material is collected and this study consisted in a survey. All participants provided consent when they replied to the first questionnaire.

## Results

Of the 272 women with false-positive findings, 179 had cancer ruled out immediately at the recall clinic (waiting time of 0 days), 46 women waited between 1 and 30 days, 36 women between 31 and 120 days, and 11 women waited more than 120 days until cancer was excluded.

**Figure 1:**
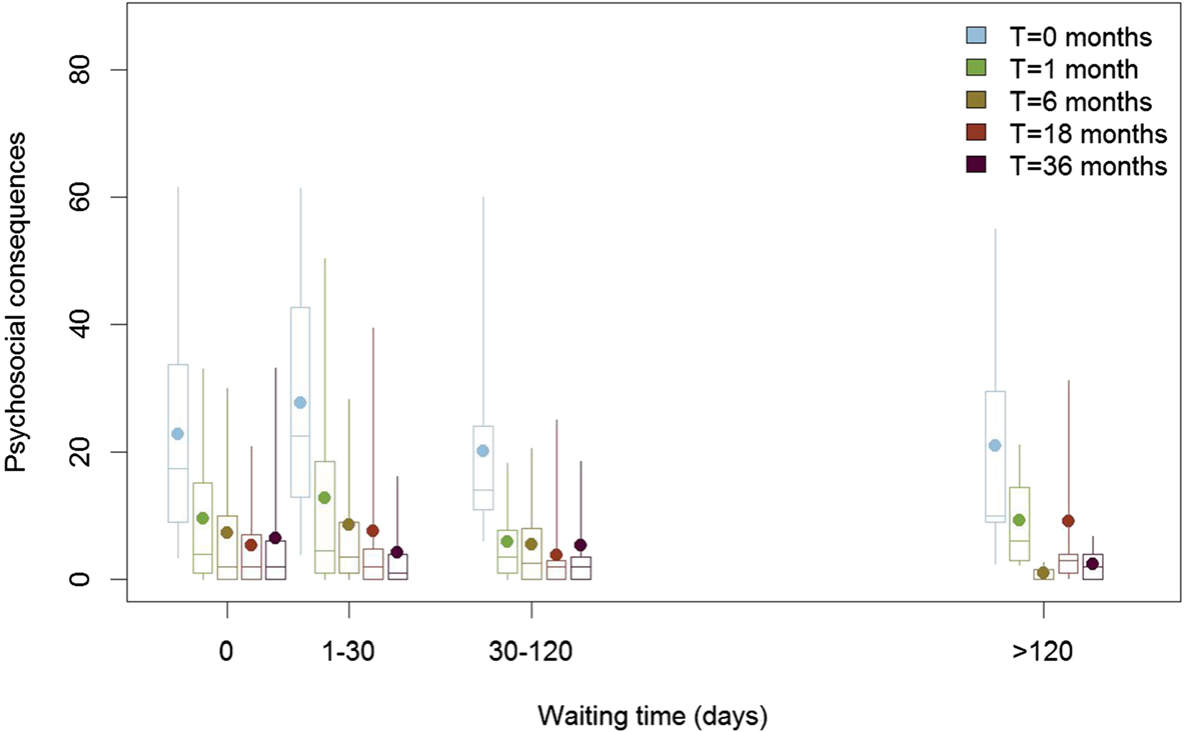
Psychosocial consequences of false-positive mammography in women who had different waiting times until cancer exclusion. For each of the groups defined by the waiting time from the day women contacted the recall clinic to final diagnosis, we assessed psychosocial consequences at five assessment points (baseline and 1, 6, 18, and 36 months after final diagnosis).

Table 1 and Figure 1 summarise the mean psychosocial consequences for each subgroup. At the baseline assessment, there were no differences in psychosocial consequences. After women were told that cancer had been ruled out, adverse psychosocial consequences decreased with time in all subgroups. Table 1 also shows that, in the 4 assessment points after cancer was ruled out, there were no statistically significant differences between women who had to wait longer for their final diagnosis (waiting times 1-30, 30-120 and > 120 days) and women who had cancer ruled immediately at the recall visit (waiting time of 0).

**Table 1:** **Differences in psychosocial consequences of false-positive mammography in women who had different waiting times until cancer exclusion**

**Time until cancer is ruled out**	**Time of assessment**				
	**Before meeting with the doctor**	**After cancer had been ruled out**			
	**0 months**	**1 month**	**6 months**	**18 months**	**36 months**
	Crude average psychosocial consequences Estimate (95% confidence interval)				
0 days	22.78	9.63	7.26	5.41	6.46
(n = 179)	(19.14 to 26.42)	(7.46 to 11.80)	(5.02 to 9.50)	(3.68 to 7.14)	(4.18 to 8.74)
1-30 days	27.65	12.87	8.61	7.57	4.17
(n = 46)	(18.51 to 36.79)	(6.18 to 19.55)	(3.03 to 14.19)	(2.10 to 13.03)	(1.20 to 7.15)
31-120-days	20.14	5.93	5.56	3.86	5.37
(n = 36)	(13.14 to 27.15)	(1.56 to 10.29)	(2.16 to 8.95)	(0.65 to 7.06)	(0 to 11.41)^†^
>120 days	21.00	9.29	1.00	9.20	2.44
(n = 11)	(4.76 to 37.24)	(3.24 to 15.33)	(0 to 2.96)^†^	(0 to 23.38)^†^	(0.59 to 4.30)
	Adjusted average difference compared with women who waited 0 days* Estimate (95% confidence interval)				
1-30 days	2.23	0.42	−1.71	−0.08	−2.67
	(-6.43 to 10.9)	(-4.39 to 5.23)	(-5.8 to 2.37)	(-3.83 to 3.67)	(-7.3 to 1.95)
31-120-days	−4.44	−3.90	−1.93	−1.35	−1.58
	(-13.38 to 4.51)	(-10.7 to 2.9)	(-6.88 to 3.03)	(-5.72 to 3.02)	(-7.12 to 3.96)
>120 days	−1.90	−0.14	−5.75	3.59	−3.67
	(-15.81 to 12)	(-9.75 to 9.46)	(-16.69 to 5.19)	(-4.82 to 12.01)	(-11.77 to 4.42)

*Positive values mean that psychosocial consequences were worse for women needing to wait longer, compared with women that had cancer excluded in the same day. The difference was adjusted for age, social class, employment, and whether the woman lived alone. ^†^The lower bound of the confidence interval was truncated at 0 since this was the lowest possible score.

## Discussion

We assessed psychosocial consequences at five different assessment points, but the interpretation of the first is different from the last four. The first consists of the assessment at the recall clinic, when women only knew that they had an abnormal screening mammography. This assessment point shows that women in the subgroups we defined for this paper were comparable at baseline. The other four assessment points were after women had been told they were free from cancer: we found no association between waiting time until cancer exclusion and the degree of psychosocial consequences at 1, 6, 18 and 36 months after cancer exclusion.

This was unexpected: qualitative studies stress that women are anguished while they wait for the results [9,10], and it was reasonable to think that the longer the wait, the worse women might feel in the long-term. A cohort study found that women that had to wait 6 or 12 months until final diagnosis were more distressed than women who had cancer ruled out after clinical mammography [17-19]; but, to the best of our knowledge, no one tried to replicate this result since. The closest comes from a cohort which included women with false-positive mammography and breast cancer. It found a non-linear effect of time on anxiety measured 7-8 months after mammography: women with very short (≤ 7 days) or very long periods (> 60 days) were more distressed than an intermediate group [13].

The main strengths of the present study are in the characteristics of the original cohort study. It used a validated condition-specific questionnaire, it was designed prospectively with repeated measurements to avoid recall bias, and it has one of the longest durations of follow-up for surveys of psychosocial harm (36 months). In addition, this study measured the main exposure of interest (waiting time) from the administrative records, which minimises recall bias.

This study’s results are limited by the small number of women included in some of the subgroups. The cohort study that suggested increased distress [17-19] had 100 women that needed to wait 6 or 12 months until final diagnosis. Our study had 11 women in the > 60 days category. Hence, lack of an association may just be due to low power. One further limitation is that we do not have data about how long women waited between receiving the recall letter and calling the screening clinic. Thus, it is possible that we misclassified women that waited some days before calling the screening clinic with a waiting time of 0. If waiting time was associated with psychosocial consequences, this misclassification would decrease the contrast between subgroups which could also explain or negative findings.

## Conclusion

This study tried to assess if there is a sub-group of women with false-positive results at particularly high risk of psychosocial harm. We did not confirm our *a priori* hypothesis: that waiting time was associated with worse long-term psychosocial consequences in women with false-positive screening mammography. However, type II error (failure to detect a true difference) might be a plausible explanation for our results.

## Abbreviations

COS-BC: Consequences of screening in breast cancer questionnaire

## References

[1] Brodersen J, Siersma VD (2013). Long-term psychosocial consequences of false-positive screening mammography. Ann Fam Med.

[2] Heleno B, Siersma VD, Brodersen J. Impact of diagnostic invasiveness on the psychosocial consequences of false-positive mammography: cohort study. Ann Fam Med. 2015; Forthcoming. 10.1370/afm.1762 PMC4427419 25964402

[3] Brett J, Bankhead C, Henderson B, Watson E, Austoker J (2005). The psychological impact of mammographic screening. A systematic review. Psycho-Oncol.

[4] Brewer NT, Salz T, Lillie SE (2007). Systematic review: the long-term effects of false-positive mammograms. Ann Intern Med.

[5] Hafslund B, Nortvedt MW (2009). Mammography screening from the perspective of quality of life: a review of the literature. Scand J Caring Sci.

[6] Salz T, Richman AR, Brewer NT (2010). Meta-analyses of the effect of false-positive mammograms on generic and specific psychosocial outcomes. Psycho-Oncol.

[7] Metsälä E, Pajukari A, Aro AR (2012). Breast cancer worry in further examination of mammography screening - a systematic review. Scand J Caring Sci.

[8] Bond M, Pavey T, Welch K, Cooper C, Garside R, Dean S, et al. Systematic review of the psychological consequences of false-positive screening mammograms. Health Technol Assess. 2013;17(13). doi:10.3310/hta17130 10.3310/hta17130 PMC4781086 23540978

[9] Thorne SE, Harris SR, Hislop TG, Vestrup JA (1999). The experience of waiting for diagnosis after an abnormal mammogram. Breast J.

[10] Lindberg LG, Svendsen M, Dømgaard M, Brodersen J (2013). Better safe than sorry: a long-term perspective on experiences with a false-positive screening mammography in Denmark. Health Risk Soc.

[11] Welch H (2004). Should I, be tested for cancer?: maybe not and here’s why.

[12] Lindfors KK, O’Connor J, Parker RA (2001). False-positive screening mammograms: effect of immediate versus later work-up on patient stress. Radiology.

[13] Haas J, Kaplan C, McMillan A, Esserman LJ (2001). Does timely assessment affect the anxiety associated with an abnormal mammogram result?. J Womens Health Gend Based Med.

[14] Brodersen J (2006). Measuring psychosocial consequences of false-positive screening results: breast cancer as an example. [Copenhagen]: University of Copenhagen.

[15] Brodersen J, Thorsen H, Kreiner S (2007). Validation of a condition-specific measure for women having an abnormal screening mammography. Value Health.

[16] Brodersen J, Thorsen H (2008). Consequences of screening in breast cancer (COS-BC): development of a questionnaire. Scand J Prim Health Care.

[17] Ong G, Austoker J, Brett J (1997). Breast screening: adverse psychological consequences one month after placing women on early recall because of a diagnostic uncertainty. A multicentre study. J Med Screen.

[18] Brett J, Austoker J, Ong G (1998). Do women who undergo further investigation for breast screening suffer adverse psychological consequences? a multi-centre follow-up study comparing different breast screening result groups five months after their last breast screening appointment. J Public Health Med.

[19] Brett J, Austoker J (2001). Women who are recalled for further investigation for breast screening: psychological consequences 3 years after recall and factors affecting re-attendance. J Public Health Med.

